# Natural Products from the Marine Sponge Subgenus *Reniera*

**DOI:** 10.3390/molecules26041097

**Published:** 2021-02-19

**Authors:** Xuelian Bai, Yang Liu, Hao Wang, Huawei Zhang

**Affiliations:** 1 College of Life and Environmental Sciences, Hangzhou Normal University, Hangzhou 311121, China; baixl2012@163.com; 2 School of Pharmaceutical Sciences, Zhejiang University of Technology, Hangzhou 310014, China; liu1395719032@163.com (Y.L.); hao_wang_3@163.com (H.W.)

**Keywords:** marine sponge, *Haliclona*, *Reniera*, endozoic microbe, natural product

## Abstract

Marine sponges are one of the prolific producers of bioactive natural products with therapeutic potential. As an important subgenus of *Haliclona*, *Reniera* sponges are mainly distributed in the Mediterranean Sea and Atlantic area, and had been chemically investigated for over four decades. By an extensive literature search, this review first makes a comprehensive summary of all natural products from *Reniera* sponges and their endozoic microbes, as well as biological properties. Perspectives on strengthening the chemical study of *Reniera* sponges for new drug-lead discovery are provided in this work.

## 1. Introduction

Marine sponges are widely distributed across oceans and represent one of the most diverse groups of primitive multicellular aquatic animals in nature. Numerous chemical investigations have indicated that this marine creature is one of the most attractive sources of precious natural products with the potential of clinical application [1]. As one of the important marine sponges found in the Mediterranean Sea and Atlantic area, *Reniera* was originally assigned as one genus and later classified to be one subgenus of *Haliclona* [2]. Morphologically, *Reniera* sponge has a soft texture and brownish-maroon epidermis, and its body is soft and fragile and looks like a compressed tree with simple digitate branches and spicules of various sizes [3]. Meanwhile, this marine sponge harbors a special arrangement of the flagellated chambers in the incurrent and excurrent canal systems [3]. To the best of our knowledge, the subgenus *Reniera* consists of at least 16 species, including *R. albescens*, *R. coccinea*, *R. cratera*, *R. fallaciosa*, *R. fascigera*, *R. fragilis*, *R. fulva*, *R japonica*, *R. lacteal*, *R. membrana*, *R. mucosa*, *R. porosa*, *R. porrecta*, *R. reticulata*, *R. sarai*, and *R. thomasii* [4]. On the basis of an extensive literature search using SciFinder and Dictionary of Natural Products databases covering up to December 2020, this review comprehensively makes an overview of all natural products from *Reniera* sponges and their endozoic microbes, as well as biological properties.

## 2. Natural Product Inventory of the Subgenus *Reniera*

Chemical studies of the marine sponge subgenus *Reniera* date back to the early 1970s. Until 2020, as many as 121 natural products had been isolated and characterized from *Reniera* sponges and their endozoic microbes. According to their chemical structures, these biomolecules are grouped into five types including alkaloid, terpenoid, polyketide, sterol, and cerebroside and ceramide, which are respectively introduced in detail as follows.

### 2.1. Alkaloids

#### 2.1.1. 3-Alkylpyridiniums

**Figure 1:**
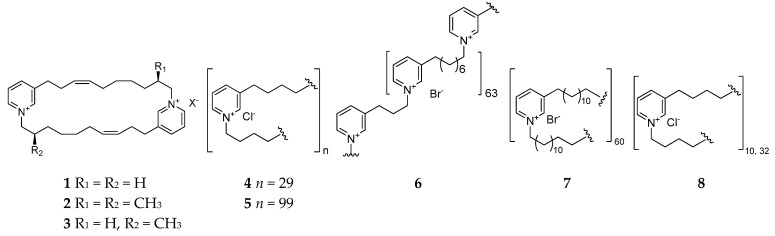
3-Alkylpyridiniums **1**–**5** from the subgenus *Reniera* and their derivatives **6**–**8.**

*Reniera* sponge-derived 3-alkylpryridiniums are inseparable dimers or polymers with various degrees of polymerization (DP) and different lengths of alkyl chains. Usually, polymeric 3-alkylpyridinium salt (Poly-APS) possesses a broad spectrum of biological properties, including a potent inhibitory effect on acetylcholinesterase and phosphatase 2A, and cytotoxic, hemoclasis, and proarrhythmogenic activity [5,6,7,8,9,10,11,12,13]. Moreover, these natural products had been found to inhibit microfouling, and the proliferation and movement of susceptible algae and biofilm bacteria [14,15,16]. The chemical study of *Reniera* sp. collected from Pemba Island (Tanzania) afforded three novel cyclic 3-alkylpyridiniums named njaoaminiums A (**1**), B (**2**), and C (**3**) (Figure 1), of which compound **2** has weak cytotoxicity against three human tumor cell lines A549 (lung carcinoma), HT29 (colon carcinoma), and MDA-MB-231 (breast) with GI_50_ values of 4.1, 4.2, and 4.8 μM, respectively [17]. Two cyclic poly-APSs (**4** and **5**) with a respective DP of 29 and 99 were separated and characterized from the Mediterranean specimen of *R. sarai* [18]. One search for the chemical synthesis of poly-APS resulted in the production of three novel analogs APS8 (**6**), APS12-2 (**7**), and APS3 (**8**), of which compound **8** is a mixture of two polymers with DPs of 10 and 32 covalently linked *N*-butyl-3-butyl pyridinium units in a 9:1 ratio. Bioassay suggested that compound **6** exhibited a toxic effect on the non-small cell lung cancer (NSCLC) tumor cell line but nontoxicity against normal lung fibroblasts [9], while **7** could cause vascular smooth muscle contraction and a decrease in arterial blood pressure and **8** could block muscle-type nicotinic acetylcholine receptors (nAChRs) [19,20].

#### 2.1.2. Quinolines and Isoquinolines

**Figure 2:**
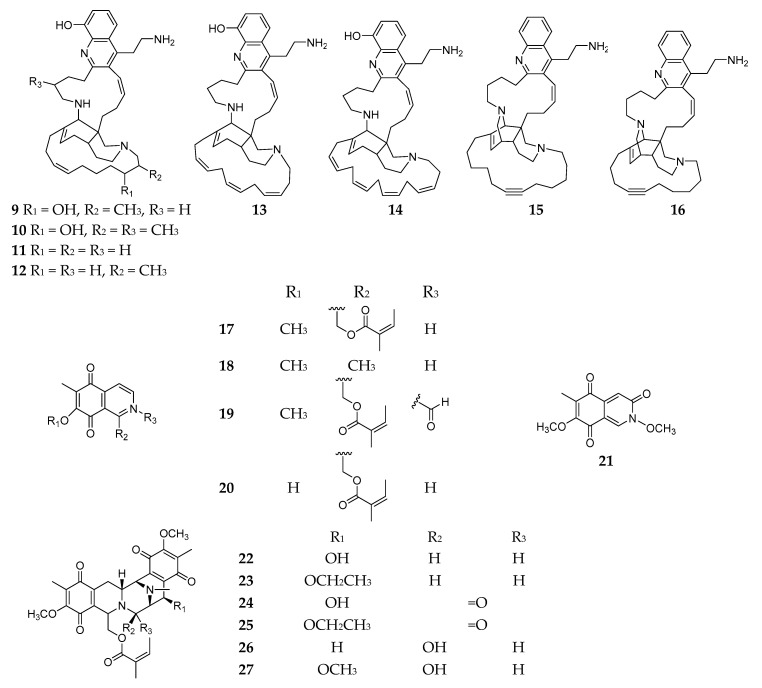
Quinolines and isoquinolines **9**–**27** from the subgenus *Reniera.*

Bioassay fractionation of the 2-propanol crude extract of *Reniera* sp. collected from the Njao area (Tanzania) afforded eight new polycyclic quinolines and njaoamines A–G and I (**9**–**16**) (Figure 2); compounds **9**–**14** demonstrated potent cytotoxicity against human tumor cell lines colon H-T29, lung A-549, and breast MDA-MB-231 with GI_50_ values ranging from 1.5 to 7.2 μM; and compound **16** had cytotoxic effect on three human tumor cell lines including MDA-MB-231 (breast), HT-29 (colon), and NSLC A-549 in the micromolar range [21,22]. Chemical analysis of the similar specimen collected in Isla Grande (Mexico) afforded nine antimicrobial isoquinolines (**17**–**25**), including five monomers renierone (**17**), 7-methoxy-1,6-dimethyl-5,8-dihydroisoquinoline-5,8-dione (**18**), *N*-formyl-1,2-dihydrorenierone (**19**), *O*-demethylrenierone (**20**), and mimosamycin (**21**), and four dimers renieramycins A–D (**22**–**25**) [23,24]. In addition, two new polycyclic isoquinoline dimers, renieramycins E (**26**) and F (**27**), were purified from another *Reniera* specimen collected from Palau [25].

#### 2.1.3. Macrocyclic Diamines

**Figure 3:**
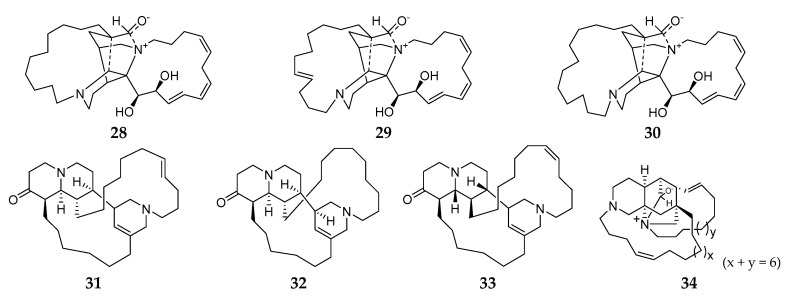
Macrocyclic diamines derivatives **28**–**34** from the subgenus *Reniera*.

To the best of our knowledge, all macrocyclic diamine-producing *Reniera* sponges were collected from the Mediterranean. Saraines A–C (**28**–**30**) (Figure 3) were obtained from the Mediterranean sponge *R. sarai* and exhibited a broad spectrum of biological activities, including insecticidal and acaricidal potency to the arthropoda *Macrosiphum euphorbiae* (Thos.), *Tetranychus urticae* Koch, and *Aedes aegypti* L.; strong inhibitory effect on *Streptococcus agalactiae* and AChE; and high hemolysis [26,27,28]. Chemical synthesis of compound **28** had first been achieved by Garg and coworkers in 2006 [29]. Isosaraine-1–3 (**31**–**33**) were three new hexahydro-quinolizin-2(6*H*)-one derivatives and their absolute stereochemistry was unambiguously determined using the modified Mosher’s method [30,31,32]. One novel macrocyclic alkaloid, misenine (**34**), was purified from unclassified *Reniera* sponge collected in the Bay of Naples (Italy) [33]. Unfortunately, no report of their bioactivity has been published until now.

#### 2.1.4. Other Alkaloids

**Figure 4:**
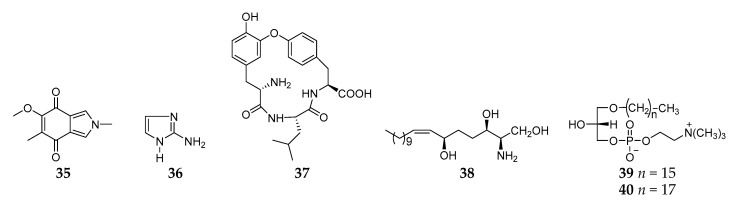
Other alkaloids derivatives **35**–**40** from the subgenus *Reniera.*

A search for antimicrobial substance(s) from an unidentified *Reniera* sponge from Isla Grande (Mexico) led to the isolation of one new isoindole-4,7-dione derivative (**35**) (Figure 4) [24], which was chemically synthesized through the cycloaddition of a nonstabilized azomethine ylide and a quinone by Parker and coworkers in 1984 [34]. Chromatography on a column of a cation exchange resin of the *n*-butanol soluble fraction from the acetone extracts of the sponge *R. cratera* afforded one simple nitrogenous compound 2-aminoimidazole (**36**) [35]. One cyclic depsipeptide renieramide (**37**) was also isolated and characterized from *Reniera* sp. No. 2115 collected on the Island of Santo (Vanuatu) [2]. Bioassay-guided fractionation of the MeOH extract of one marine sponge *Haliclona* (*Reniera*) sp. collected off Ulleung Island (Korea) led to the discovery of a new sphingosine (**38**) together with two lysophosphatidylcholines (**39** and **40**), which exhibited moderate cytotoxicity against a panel of five human solid tumor cell lines including A549, SK-OV-3, SK-MEL-2, XF498, and HCT15 [36].

### 2.2. Terpenoids

**Figure 5:**
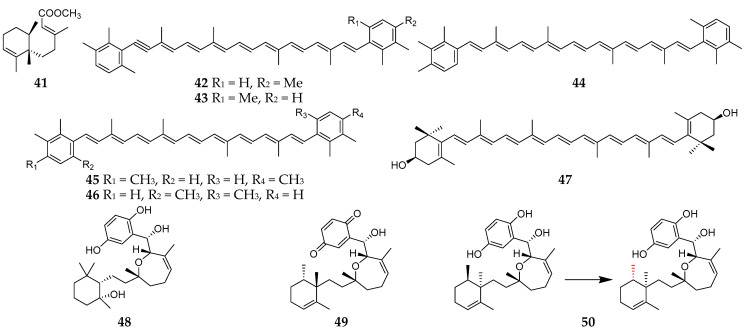
Terpenoids **41**–**50** from the subgenus *Reniera.*

Structurally, most of terpenoids produced by *Reniera* sponges are tetraterpenes except the sesquiterpenoid fulvanin 1 (**41**) (Figure 5) [37]. Interestingly, these tetraterpenes are carotenoid analogs, including acetylenic carotenoids (**42**–**43**), renieratene (**44**), isorenieratene (**45**), and renierapurpurin (**46**) from *R. japonica* [38,39]. It is noteworthy that two symbiotic strains, *Flexibacter* sps. DK30213 and DK30223, isolated from *R. japonica*, were found to produce zeaxanthin (**47**), which is one of the commonly used antioxidant agents [40]. Two new cytotoxic meroditerpenes, halioxepine B (**48**) and halioxepine C (**49**), along with halioxepine (**50**), were isolated from two Indonesian sponges of the genus *Haliclona* (*Reniera*) and structurally determined by QM/NMR-DFT (quantum mechanics combined with nuclear magnetic resonance parameters calculated by density functional theory approximations) analysis [41]. It is noteworthy that compound **50** had first been synthesized and the absolute configuration at position C15 was revised as *S* [42].

### 2.3. Polyketides

#### 2.3.1. Aromatic Polyketides

**Figure 6:**
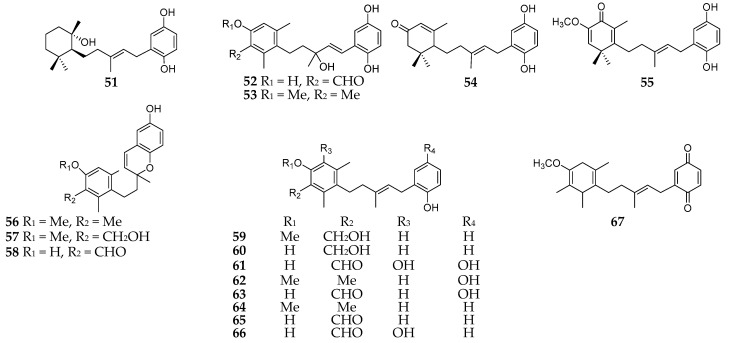
Aromatic polyketides derivatives **51**–**67** from the subgenus *Reniera.*

Polyketides are one of the major groups of *Reniera*-derived secondary metabolites, such as aromatic and aliphatic polyketides. As many as eighteen aromatic polyketides (**51**–**67**) (Figure 6) had been separated from *R. fulva* and *R. mucosa*, which were respectively collected from the Egadi Islands (Italy) and Tarifa Island (Spain) [37,43]. Compounds **52**, **53**, **56**, and **59** possessed in vitro cytotoxicity against P388 mice lymphoma, A549 human lung carcinoma, HT29 human colon carcinoma, and MEL28 human melanoma cell lines with the same ED_50_ values of 5 mg/mL [43]. Moreover, compound **59** exhibited a moderate inhibitory effect on DHFR (dihydrofolate reductase) with an ED_50_ value of 3 mg/mL. At the concentrations from 10^−4^ to 10^−8^ M, compounds **61** and **63** were shown to be cytotoxic to the NCI-H522 nonsmall lung cancer cell line and CCRF-CEM leukemia cell line, while **54** had more selective cytotoxicity against the latter [37].

#### 2.3.2. Aliphatic Polyketides

**Figure 7:**
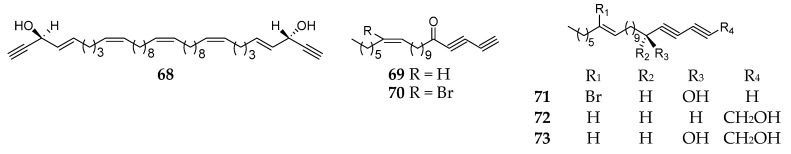
Aliphatic polyketides **68**–**73** from the subgenus *Reniera.*

Linear alkynols and alkynones are the most common aliphatic polyketides detected in the marine sponge *R. fulva*. Fulvinol (**68**, Figure 7), a new long-chain diacetylenic compound, was purified from *R. fulva* collected at Algeciras Bay (Spain) and found to possess an inhibitory effect on P-388 mouse lymphoma, A-549 human lung carcinoma, HT-29 human colon carcinoma, and MEL-28 human melanoma cell lines with the same ED_50_ values of 1 μg/mL [44]. One search for secondary metabolites of *R. fulva* from the Mediterranean Sea resulted in the isolation of five new acetylenic compounds including debrorenierin-1 (**69**), renierin-1 (**70**), lb-dihydrorenierin-1 (**71**), renierin-2 (**72**), and 18-hydroxyrenierin-2 (**73**) [45].

#### 2.3.3. Other Polyketide

**Figure 8:**
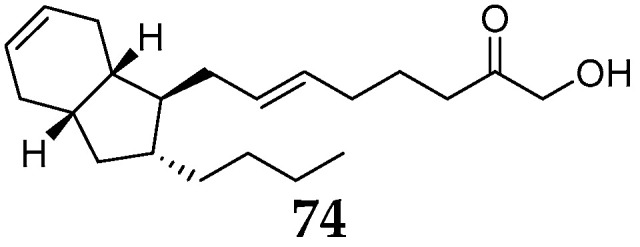
Other polyketide **74** from the subgenus *Reniera.*

One new bicyclic eicosanoid named mucosin (**74**) (Figure 8) was detected in the acetone extracts of *R. mucosa* samples, which had been collected in different areas including Blanes (Spain), Grotte de Jarr (France), Massalubrense, and Procida (Italy) [46]. Interestingly, this metabolite contains an unusual bicyclo [4.3.0] nonane skeleton with equilibrium of normal physiology in mammalian systems.

### 2.4. Sterols

**Figure 9:**
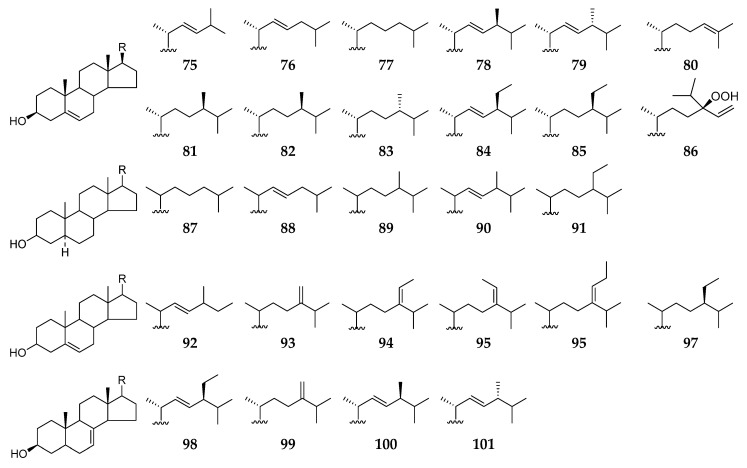
Sterols **75**–**101** from the subgenus *Reniera.*

To date, a total of 27 sterol derivatives (**75**–**101**) (Figure 9) have been obtained and characterized from *Reniera* sponges. Chemical analysis of one unidentified sample (#063176) deposited at California Academy of Sciences Museum afforded eleven sterols (**74**–**85**) [47], and another specimen (*R. sarai*) collected from the Bay of Naples (Italy) resulted in the isolation of ten sterols (**87**–**96**) [48]. Using vacuum liquid chromatography (VLC), flash column chromatography, and preparative thin-layer chromatography (PTLC), *β*-sitosterol (**97**) was purified from ethyl acetate extract of *Haliclona* (Reneira) *fascigera* sponge (SPV06/12/13) collected off Samalona Island (Indonesia) [49]. Along with three alkaloids (**38**–**40**), six sterol analogs **77**, **86**, and **98**–**101** were separated from the MeOH extract of the sponge *Haliclona* (*Reniera*) sp. J01U-6 from Ulleung Island (Korea) [36].

### 2.5. Cerebrosides and Ceramide

**Figure 10:**
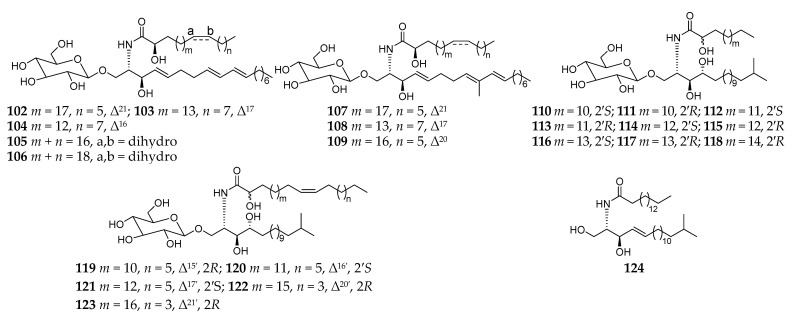
Cerebrosides **102**–**123** and ceramide **124** from the subgenus *Reniera.*

Cerebrosides are a group of glycosphingolipids consisting of a glucose or galactose residue attached to a ceramide moiety containing one sphingoid base and an amide-linked long fatty acyl chain. These amphipathic biomolecules are important components of tissues and organs in organisms and possess a broad spectrum of biological functions such as antifungal, antitumor, antiviral, an inhibitory effect on histidine decarboxylase, and cytotoxicity [50]. Chemical analysis of the *n*-hexane layer of the MeOH extract of the same specimen *Haliclona* (*Reniera*) sp. J01U-6 collected off the coast of Ulleung Island (Korea) afforded twenty-one new cerebrosides (**101**–**111**, **113**–**122**) together with one known analog (**112**), which possess unprecedented unsaturated or saturated long (C_15_–C_28_) alkyl chains (Figure 10) [51,52]. This was the first report on the isolation of isomeric pairs of glucocerebrosides containing saturated C_15_ and C_19_ acyl chains. Lately, the structural determination of compounds **109**–**112**, **115**, **116**, **120**, and **121** were well established using fast atom bombardment mass spectrometry (FAB-MS) in positive-ion mode by Hong and coworkers [53]. In addition, one ceramide (**123**) was separated from the same sponge SPV06/12/13 collected off Samalona Island, and its absolute structure was determined by HyperChem computational techniques [49].

## 3. Conclusions and Perspectives

The marine sponge subgenus *Reniera* is one of the most prolific sources of natural products possessing various chemical structures and biological properties, such as cytotoxic poly-APS derivatives, insecticidal and acaricidal sarains A–C (**28**–**30**), and antioxidant zeaxanthin (**47**). In the recent decade, however, few reports on biological and chemical studies of *Reniera* sponge had been published. In comparison with those of other marine sponge genera such as *Agelas* [54] and *Phyllospongia* [55], chemical investigations of *Reniera* sponges seem to be less intensive. Therefore, great efforts should be made to carry out global resource surveys and collections of *Reniera* sponges and chemical studies using hyphenated technology, such as GC-MS and LC-MS-NMR. Furthermore, more attention should be paid on genome mining and the chemical study of symbiotic microorganisms of *Reniera* sponges as these microbes are potential producers of bioactive secondary metabolites originally derived from their hosts [56,57,58].
